# More Dialectical Thinking, Less Creativity? The Relationship between Dialectical Thinking Style and Creative Personality: The Case of China

**DOI:** 10.1371/journal.pone.0122926

**Published:** 2015-04-09

**Authors:** Hui Liu, Fei-xue Wang, Xiao-yang Yang

**Affiliations:** Department of Psychology, Sun Yat-sen University, Guangzhou, China; University of Westminster, UNITED KINGDOM

## Abstract

People use dialectical thinking to be holistic, reconcile contradictions, and emphasize changes when processing information and managing problems. Using a questionnaire survey, this study examined the relationship between dialectical thinking and creative personality in the Chinese culture, which encourages a holistic and collective thinking style. Undergraduates majoring in different subjects and adults in different professions were surveyed. The results showed that 1) compared with undergraduates majoring in art and adults from the design industry, undergraduates majoring in other disciplines significantly showed the least creative personality; 2) the highest score for dialectical thinking was found in the group of undergraduates who majored in other disciplines, followed by the adult group, and the undergraduates majoring in art had the lowest score; and 3) A negative relationship between dialectical thinking and creative personality was found mostly in the UMA group. The limitations of this study and suggestions for future research are discussed.

## Introduction

Creativity, the generation of work that is both novel and appropriate [1], is an important skill for coping with many different circumstances in life [2] and for organizations and societies [3]. It is one of the most important catalysts of social development and improvement. The important role of creativity in human progress and civilization is likely one reason why scholars across disciplines have long been interested in understanding what creativity is, how creative thinking occurs, and how such thinking can be fostered [4].

Creativity research in psychology, as one primary object of cross-disciplinary research, has been conducted from different perspectives. There are three main trends in psychological research on creativity: some work focuses on how to measure creativity products and personal accomplishments [5–7]. Other work has explored the cognitive and motivational processes that lead to creative ideas [8–11]. The third research trend, which examines the relationship between individuals’ personality and creativity [4] [12–15], has long drawn the attention of numerous researchers. Fruitful research findings in this area allow us not only to understand the apparent personality traits that can be related firmly with a person who shows high levels of creativity but also to go further into the causative factors and differences in cultural background.

Raised in a typical East Asian culture, Chinese people follow a type of holistic and collective thinking that has created traditional Chinese thinking styles in every corner in their daily lives. This style is also called dialectical thinking. Nisbett et al. [16] [17] indicate that, in contrast to the analytical thinking style of Western culture, East Asian culture, which is typically represented by China, can be distinguished easily by its holistic thinking style, and dialectical thinking is the core of this thinking style. Western culture is characterized by individualism, attaching importance to independence, autonomy, and creativity, whereas Eastern culture emphasizes collectivism and values obedience, cooperation, duty, and authority. Previous empirical research has also found that, compared with collectivism, individualism is more conducive to creativity [18].

In terms of this argument, an individual’s thinking style has a close relationship with creativity, and as another type of individual difference, thinking style is also affected by certain cultural and social circumstances. In other words, some types of cultures improve creativity by means of thinking styles, but other types of cultures do not. Based on this understanding, we are interested in the relationship between thinking style and creative personality in Chinese culture. We want to explore whether Chinese culture (or an East Asian culture) facilitates creativity.

### Creative Personality

Although people always focus on the production, cognitive process, and circumstances of creativity, there are many studies of people who are considered to be creative. Numerous studies have been conducted in the past several decades. Qian, Plucker, and Shen [19] proposed three apparent themes in the research of the creative personality. The first theme is the individual differences between creative people. A considerable body of literature touches on the individual differences in personality traits [20] [21]. For instance, Tardiff and Sternberg [22] proposed 19 creative personality traits by summarizing other psychologists’ views. Feist [13] conducted a meta-analysis of personality in scientific and artistic creativity, concluding that “in general, creative people are more open to new experiences, less conventional and less conscientious, more self-confident, self-accepting, driven, ambitious, dominant, hostile, and impulsive” (p. 290).

The second theme is the construct of creative personality. After reviewing more than 100 articles that investigated creative personality characteristics, Treffinger, Young, Selby, Shepardson, and Florida [23] concluded that creative individuals can be divided into two different types. The first type of creative individuals was characterized by traits such as openness to experience, risk-taking, playfulness, intuition, and curiosity, whereas the second type displayed traits such as independence of thought, introspection, an internal locus of control, persistence, self-confidence, and hard-working tendencies.

The third theme is longitudinal studies about creative personality, which always adopt a developmental research method. For example, Nie and Zheng [24] proposed different developmental stages for middle school students’ creative personalities. Wang [25] concluded that after the seventh grade, adolescents’ creative tendencies showed consistently decreasing trends.

All of these findings constitute our knowledge of creative personality traits. However, the role of circumstances, especially culture and other environmental factors, was seldom noted in these research trends. Simonton [4] [15] [26–28] used a historiometric method to investigate thousands of eminent creators from different fields and domains in Western history. He concluded that social environments can have nurturing (or inhibitory) effects on the development of creativity. In his research, social environments consist of cultural conditions, economics, politics, and other important environmental factors. To investigate the effect that a social environment may have on an individual’s creative personality, the present study attempts to take the first step in research about the influence of culture on creative personality in East Asian culture, using Chinese culture as an example.

### Culture and Creativity

A culture is the specific part of a society or civilization related to its beliefs, way of life, or art. Culture is considered important for the development of human society and humans’ behavior and mind [29]. Csikszentmihalyi [30] has proposed that creativity is more of a cultural and social phenomenon than merely a mental process. Simonton [27] [31] has suggested that the cultural context, such as the economic, political, social, and cultural conditions, can have a significant effect on the contributions and the evaluation of those contributions within a given field of activity. Sternberg and Lubart [32] proposed that the effects of culture on creativity can manifest in four ways: the way creativity is defined, the process of creativity, the impact of creativity in one domain on others, and the extent to which creativity is nurtured. A consensus in these studies is that cultural conditions can easily be connected with creativity. However, can we go any further into this relationship? The theory with respect to individualism and collectivism [33] or with respect to an independent-interdependent perspective [34] may provide possible explanations.

For example, as proposed by Markus and Kitayama [34], people in Western cultures (such as European and American cultures) usually hold an independent perspective of the self, in which one’s behavior is organized and made meaningful largely in reference to one’s own internal thoughts, feelings, and actions rather than the thoughts, feelings, and actions of others. By contrast, people in Eastern cultures (such as Chinese and Japanese cultures) hold an interdependent perspective of the self, in which people are usually motivated to find a way to fit in with relevant others and become part of various interpersonal relationships.

Niu and Sternberg [18] designed two studies to compare artistic creativity between American and Chinese college students. The results of this study seem to support the hypothesis that an independent self-oriented culture better encourages the development of artistic creativity than an interdependent self-oriented culture. This conclusion can also be drawn from research on scientific creativity. A similar cross-cultural study [35] compared the scientific creativity performance between 2,277 English and Chinese adolescents aged 11 to 18. They found that English adolescents show markedly higher levels of scientific creativity than Chinese adolescents, especially in creative imagination, product improvement, and overall scientific creativity. Zha, Walczyk, Griffith-Ross, Tobacyk, and Walczyk [36] explored the relationship between culture and creative potential in highly educated adults. They surveyed 55 American and 56 Chinese doctoral students to assess their creative potential and their sense of individualism or collectivism. Americans displayed significantly higher scores on creative potential than the Chinese. As expected, Americans showed greater individualism, but the Chinese were more collectivistic. Another study used a German and Chinese sample and compared the performances of artistic creativity of the participants of each culture. The results also revealed that the German participants (Caucasian Germans and Asian Germans) produced more creative and aesthetically pleasing artwork than did their Chinese counterparts (Chinese studying abroad and domestic Chinese) and that there were no significant subgroup differences in creative performances, i.e., no differences between the two German groups and no differences between the two Chinese groups [37]. All of these studies seemingly indicate that the Chinese cultural background is an obstacle to creativity development. However, the factor(s) under this cultural condition that significantly impede the expression of creativity in Chinese people especially require further investigation.

### Dialectical Thinking

Several studies have indicated that people in different cultures have different systems of thought. Nisbett et al. [16] [17] found East Asians to be *holistic*, attending to the entire field and assigning causality to it, making relatively little use of categories and formal logic, and relying on “dialectical” reasoning, whereas Westerners are more *analytic*, focusing primarily on the object and the categories to which it belongs and using rules, including formal logic, to understand its behavior. In other words, compared with analytical thinking, people with holistic thinking styles are oriented toward continuity and relationships. Their explanations and attributions are embedded within a large context of which one is an interdependent part. The classifications are based more on relationships and similarities than on rules. The reasoning process relies on experiential knowledge more than formal logic, and dialectical thinking is regarded as an effective tool for managing seeming contradictions—retaining basic elements of opposing perspectives by seeking a “middle way”.

Dialectical thinking is one of the most important parts of holistic thinking. After exploring dialectical thinking by comparing lay Chinese reactions to apparent contradictions with those of lay Americans, Peng and Nisbett [38] differentiated Chinese dialectical thinking from Western traditional dialectical reasoning and defined it as “Naive Dialecticism”. As a distinguishable cognitive style, three principles were identified as a thorough understanding of this dialectical thinking: the principle of change, the principle of contradiction, and the principle of relationship or holism. 1) The principle of change states that reality is always changing. It is not so much a phenomenon as a *process*. 2) The principle of contradiction holds that reality is full of contradictions. It is not precise or cut-and-dried. Contradiction is constant and coexists in everything. 3) The principle of relationship or holism is proposed based on the principles of change and contradiction. It is regarded as the essence of dialectical thinking. There is nothing isolated and independent; everything is connected. If people really want to know something fully, they must emphasize its relationships, i.e., how it affects and is affected by everything else.

By contrast, Western thought emphasizes three different principles: the law of identity, the law of noncontradiction, and the law of the excluded middle. 1) The law of identity holds that everything is what it is; thus, A equals A. In other words, everything must be identical with itself. Everything must stand, regardless of context. 2) The law of noncontradiction declares that nothing can be true and false simultaneously; thus, A cannot equal not-A. 3) The law of the excluded middle expresses the rule that any statement is either true or false; thus, it is either A or B, and it is not A and B. From this perspective, there is no “middle way” [38].

In summary, in this study, dialectical thinking can be defined as a thought process that people employ to be holistic, reconcile contradictions, and emphasize changes when processing information and managing problems.

### Dialectical Thinking and Creativity

As noted above, Peng and Nisbett found that, when managing seeming contradictions, the Chinese preferred dialectical thinking, whereas American participants polarized their views [38]. In the past fifteen years, there has been mounting evidence suggesting that East Asians’ naive dialecticism affects their psychological processes in fundamental ways. Researchers found that East Asians are often more tolerant of the coexistence of opposing traits, emotions, and attitudes within themselves [39–41]. Because culture influences human cognition in many areas and there are many differences in cognition across cultures, the relationship between dialectical thinking and creative processes needs to be reevaluated [42]. This study was inspired by and based on this point of view.

Almost all previous studies have supported the idea that dialectical thinking is positively associated with creativity. Arlin [43] argued that a problem-finding stage of cognitive development could be the opposite side of the same coin as dialectical operations, such that “dialectical operations may be the functional dynamic of creative thought” (p. 250). Her later studies empirically confirmed this positive relationship [44] [45]. Similarly to Arlin et al., Benack, Basseches, and Swan [46] suggested that dialectical thinking is a cognitive process and that it can lead to breaking sets, paying attention to and synthesizing contradictions, being aware of the complex relationships among things, and understanding that one’s own thoughts will change. All of these characteristics, in turn, lead to increased creativity. Rosenberg [47] interviewed twenty-two Nobel laureates in the fields of chemistry, physics, and medicine and physiology from Europe and the United States. The interviews focused on in-progress creative work. Based on the results of the interviews, he proposed a *Janusian process* (“actively conceiving multiple opposites or antitheses simultaneously”) in scientific creativity, whereby the creator goes through several phases, including immersion, focusing, antithesis, and elaboration. All of these phases were viewed as constituting the creative process, and all of these phases showed dialectical characteristics.

A more comprehensive and in-depth study examined the association between naive dialectical thinking, which was regarded as the typical dialectical thinking in Eastern Asian culture, and creativity [42]. Two correlational studies and one experimental study revealed a complex pattern of results: for Caucasian participants, when problems had a low level of inherent contradiction, naive dialectical thinking decreased originality in problem finding tasks, whereas for tasks with a higher level of contradiction, there was no effect or a slightly positive effect. Due to the sampling and experimental details, this research suggests that the relationship might be culture-, task-, and process-specific. Though Paletz and Peng’s research deepened the understanding of the relationship between dialectical thinking and creativity [42], these relationships were found only for Caucasian participants and not for Asian or Asian-American participants.

In this study, we focus mainly on creative people in East Asian culture, attempt to capture the features of creative talents from a dialectical perspective, and briefly analyze the relationship between dialectical thinking and creative personality. Overall, the rationales for this study are as follows:

First, in regard to creativity, we need to know more about cultural differences. In the context of East Asian culture, a holistic, dialectical thinking style is fundamentally related to collectivism. Nevertheless, the Chinese have also proven their creativity, for example, the four great inventions of ancient China (compass, papermaking, movable type printing press, and gunpowder). No one can ignore Chinese historical creativity. What, then, is the relationship between dialectical thinking and creativity in East Asian culture? On the one hand, almost all of the previous studies were mainly conducted with the participants from Western cultures [44] [45] [47]. Even if participants from other cultures were recruited, they were regarded as a counterpart [37], [42]. On the other hand, most studies concluded that dialectical thinking is positively related to creativity, except for one study, which found that the relationship is culture-specific [42]. Obviously, researchers have not yet reached a consensus. Exactly what, then, is the relationship between culture and creativity? Answering this question is our research interest and the basis of our study. To that end, we try to dig more deeply and provide more evidence by using only Chinese participants in this study. The present study is just the first step in a series of studies that aim for a comprehensive and in-depth understanding of creative people in different cultures.

Second, the factors of personality should not be ignored. Previous studies have shown the relationships among dialectical thinking, creative problem finding, and problem solving [42][45][46][48]. However, few investigations and empirical studies have directly addressed the relationship between dialectical thinking and creative personality. Moreover, it is well established that creative personality is one of the main trends of research on creativity [13][19] [23]. Our understanding of creative personality has been enriched and constructed very well in the past several decades by enormous research achievements in the field. Based on these previous arguments and studies, we have sufficient reason to combine dialectical thinking with creative personality.

According to Paletz and Peng’s conclusion [42], the relationship might be culture-, task-, and process-specific. In this study, we recruited adults from different professional fields and undergraduates with different majors. Through this sampling process, Paletz and Peng’s study was replicated on a domain level. Thus, we established the following predictions:

Hypothesis 1: The participants will be differentiated in dialectical thinking and creative personality according to their occupation and major.

Because of the inconsistency of previous conclusions [42] [44] [45] [47], a research question should be posed: Does dialectical thinking style encourage creativity through a positive influence on creative personality? In this study, because the culture-specific viewpoint in former studies [42] is more ecological and more evidential, we assumed that a different relationship does exist between naive dialectical thinking and creative personality in East Asian culture. Based on this rationale, a negative connection was predicted and tested:

Hypothesis 2: In Chinese culture, naive dialectical thinking will be negatively related to creative personality.

## Methods

### Procedure

To estimate the difference in creativity and dialectical thinking in relation to college majors and occupational fields, we recruited subjects from several companies and different college departments. Then, their creative personality was rated using a self-reported questionnaire. Study participants also rated their own dialectical thinking using another dialectical thinking survey instrument. After the data collection, the relationship between dialectical thinking and creative personality and the differences between the groups were analyzed.

### Participants

A total of four hundred eighty-nine participants were selected through a convenient sampling method. The participants consisted of three groups according to their profession and major: undergraduates majoring in art (UMA), undergraduates majoring in other disciplines (UMO), and the adult group (AG). 1) The UMA group included 84 undergraduates from a college of art in Kunming (36 majoring in orchestral performance, 48 majoring in animation), and their average age was 19.46, SD = 0.975. 2) The UMO group included 203 undergraduates majoring in non-art disciplines who were recruited from a college in Guangzhou (86 majoring in economics, 75 majoring in Chinese language and literature, 23 majoring in bioengineering, 14 majoring in food engineering, and 5 majoring in packaging engineering). The average age was 20.06, SD = 1.227. 3) The AG group contained 202 adults from arts-related professions (95 were members of a philharmonic orchestra in Guangzhou, 90 were designers from an architectural design institute in Kunming, and 17 were designers for a game website in Shanghai). Their average age was 27.84, SD = 3.707. All of the participants in this study were ethnically Chinese.

### Ethics Statement

An informed consent form for participation in this creativity and dialectical thinking style study was signed by each participant before each survey process. Participants received another debriefing form after they finished the questionnaires. Before the study began, we reported the entire research plan, including these two forms, to the institutional review board of the Department of Psychology at Sun Yat-sen University and received approval from the board.

### Measures

#### Dialectical thinking

In Paletz and Peng’s study [42], the participants answered the 32-item Dialectical Self Scale as the measure of naive dialectical thinking [41]. In this study, to measure naive dialectical thinking more precisely and innovatively, we chose another questionnaire developed by native Chinese psychologists named the “Zhong-yong Practical Thinking Scale” (also called the “9-item Zhong-yong Thinking Scale”) [49]. The zhong-yong thinking style, defined as the careful consideration of things from different aspects and the performance of appropriate behaviors to account for the whole situation [50] [51], has almost the same conceptual structure and meanings as naive dialectical thinking in Chinese culture. Therefore, the Zhong-yong thinking scale is usually used to examine dialectical thinking in China [52]. This questionnaire has a total of nine items. Each item is composed of two opposite narrative sentences that represent dialectical and less-dialectical thinking, respectively. For instance, agreeing that “it is important to live in harmony with people around you” represents a dialectical thinking style, and agreeing that “sometimes you must go ahead regardless to strive for vindication” represents a less-dialectical thinking style. The responses of this questionnaire include two steps. In step 1, after reading two narrative sentences of an item, the subject chooses the one he or she likes better; in step 2, the subject rates his or her choice on a 6-point Likert scale from 1 (strongly disagree) to 6 (strongly agree). We used a 6-point instead of a 5- or 7-point scale because Chinese participants always prefer choosing the “middle way” (score 3 or 4), which would bias the results toward dialectical thinking. Regarding scoring, if the respondents chose a dialectical sentence in step 1, then the final score of that item was the same as the chosen score; however, if a less-dialectical sentence was chosen in step 1, the final score of that item equaled 6 minus the chosen score. The total score of the nine items represented the level of a respondent’s dialectical thinking. Yang used this questionnaire to survey 783 adult citizens; in his study, the Cronbach’s α was 0.721 [53]; Zhou, Liao, and Su sampled 406 employees from private enterprises in Taiwan, and the Cronbach’s α reported in their study was 0.73 [54].

#### Creative personality

Song developed an undergraduate creative personality scale [55]. After successful Exploratory and Confirmatory Factor Analyses, this scale finally consisted of four dimensions: imagination, perseverance, insight, and exploration. This scale is a 5-point Likert scale that ranges from 1 (strongly disagree) to 5 (strongly agree). In Song’s study, she sampled 939 undergraduates from four universities in Zhengzhou, China. The test-retest reliability of this scale is 0.872, the split-half reliability is 0.899, and the Cronbach’s α is 0.898. The criterion-related validity of this scale is 0.62 (p<0.01). All of these indexes are acceptable. The description of each item is suitable not only for undergraduates but also for average adults. Therefore, all of the participants in our study responded to this questionnaire. In our study, the Cronbach’s α of the scale was 0.732, which is also acceptable.

The questionnaires were administered to the participants in groups based on their major or profession. At the beginning, a researcher read the instructions and emphasized that the data would be kept confidential. Then, the respondents began to respond in pen and paper form independently. After data collection, we employed SPSS 16.0 for the data arrangement and analysis. Descriptive statistics, correlation, t-test, and ANOVA analyses were applied in turn.

## Results

First, the descriptive statistics (*M±SD*) were analyzed for the different sample groups and for the sample as a whole to collect basic data. Table 1 shows the descriptive statistics. To explore the differences among these three groups more deeply in terms of dialectical thinking and creative personality, two kind of analysis of variance were conducted separately. First, due to the internal correlation of different personality constructs, which showed in the personality questionnaire, a multivariate analysis of variance (MANOVA) with creative personalities as dependent variables was performed. The results of multivariate tests indicate that there were statistically significant differences among groups (*Wilks’ Lambda* = 0.950, *F* = 3.117, *df* = 8, *p* = 0.002, *partial Eta squared* = 0.025). Second, based on the results of MANOVA, one-way ANOVA was conducted six times to assess the respective dimensions of creative personality, total score of creative personality, and dialectical thinking. Significant differences were found through these analyses in three constructs, the total score of creative personality, and dialectical thinking except insight (see Table 2).

**Table 1:** Composite scores of different groups on dialectical thinking and creative personality (*M±SD*).

	UMA (*N* = 84)	UMO (*N* = 203)	AG (*N* = 202)	Total
**DT**	38.65±5.91	41.88±6.65	40.45±7.01	40.73±6.77
**Imagination**	29.87±5.28	26.77±5.59	28.49±5.74	28.01±5.71
**Perseverance**	25.67±4.26	24.35±4.60	25.47±4.51	25.04±4.53
**Insight**	17.77±3.21	16.99±3.48	17.40±3.44	17.29±3.42
**Exploration**	15.10±2.45	14.04±3.38	14.77±2.64	14.52±2.97
**Total CP**	88.40±10.85	82.15±13.06	86.12±12.01	84.87±12.49

Note. DT = dialectical thinking; UMA = undergraduates majoring in art; UMO = undergraduates majoring in other disciplines; AG = adult group; CP = creative personality (the same definitions are used in subsequent tables)

**Table 2:** Results of ANOVA of different groups on dialectical thinking and creative personality (*N* = 489).

	*df*	*F*	*Sig*.	*Partial Eta squared*
**DT**	2	7.222	0.001	0.029
**Imagination**	2	10.341	0.000	0.041
**Perseverance**	2	4.098	0.017	0.017
**Insight**	2	1.731	0.178	0.007
**Exploration**	2	4.971	0.007	0.020
**Total CP**	2	9.505	0.000	0.038

A Least Significant Difference test was used for the post hoc test. The post hoc test revealed more details about the group differences. 1) A significant main effect of dialectical thinking was found among the three groups. The results showed that in dialectical thinking, the UMO group was significantly higher than the AG (*p* = 0.040); AG was significantly higher than the UMA group (*p*<0.001); And the UMO group was also significantly higher than the UMA group (*p* = 0.032). 2) A significant main effect of imagination was found. The post hoc test suggested that the UMA group was significantly higher than the UMO group (*p*<0.001); The AG group was significantly higher than the UMO group (*p* = 0.002); and no significant difference was found between the UMA and AG groups (*p* = 0.058). 3) A significant main effect of perseverance was found. The post hoc test showed that the UMA group was significantly higher than the UMO group (*p* = 0.025); the AG group was significantly higher than the UMO group (*p* = 0.013); and no significant difference was found between the UMA and AG groups (*p* = 0.731). 4) No significant main effect of insight was found. 5) A significant main effect of exploration was found. The post hoc test showed that the UMA group was significantly higher than the UMO group (*p* = 0.006); the AG group was significantly higher than the UMO group (*p* = 0.014); and no significant difference was found between the UMA and AG groups (*p* = 0.391). 6) On the total creative personality scores, a significant main effect was found. The UMA group was significantly higher than the UMO group (*p*<0.001); The AG group was significantly higher than the UMO group (*p* = 0.001); and no significant difference was found between the UMA and AG groups (*p* = 0.152).

In summary, these results indicated that the UMA and AG groups shared similar features on creative personality, not only on the four dimensions of creative personality but also on the total score. Nevertheless, their scores were all significantly higher than the UMO group, except on the dimension of insight. Given that the domain of art and the domain of design shared some common traits in creativity [56], it may demonstrate a domain-specific nature of creative personality. Notably, the results of the difference in their dialectical thinking were completely opposite. The UMO group had the highest score on dialectical thinking, followed by the AG group, and the UMA group had the lowest score. Did this result indicate a negative relationship between dialectical thinking and creative personality?

In addition to the descriptive statistics of dialectical thinking and creative personality and the differences among groups, what we are interested in is the relationship between the two main variables in this study. A correlation analysis between dialectical thinking and creative personality was conducted on the sample as a whole (see Table 3) and on the three sample groups separately (see Tables 4, 5, and 6).

**Table 3:** Correlations between dialectical thinking and different dimensions of creative personality (*N* = 489).

	DT	Imag	Perse	Insight	Explo	Total CP
**DT**	1	-	-	-	-	-
**Imag**	-.132**	1	-	-	-	-
**Perse**	-.063	.315**	1	-	-	-
**Insight**	-.103*	.465**	.475**	1	-	-
**Explo**	-.078	.521**	.313**	.347**	1	-
**Total CP**	-.130**	.823**	.711**	.741**	.684**	1

Note. DT = dialectical thinking; Imag = imagination; Perse = perseverance; Explo = exploration; CP = creative personality (the same definitions are used in subsequent tables).

**p<0.01.

*p<0.*05* (two-tailed test).

**Table 4:** Correlations between dialectical thinking and different dimensions of creative personality: UMA group (*N* = 84).

	DT	Imag	Perse	Insight	Explo	Total CP
**DT**	1	-	-	-	-	-
**Imag**	-.273*	1	-	-	-	-
**Perse**	-.282**	.298**	1	-	-	-
**Insight**	-.323**	.452**	.509**	1	-	-
**Explo**	-.151	.249*	.134	.219*	1	-
**Total CP**	-.374**	.794**	.718**	.766**	.464**	1

**p<0.*01*;

*p<0.*05*. (two-tailed test)

**Table 5:** Correlations between dialectical thinking and different dimensions of creative personality: AG group (*N* = 202).

	DT	Imag	Perse	Insight	Explo	Total CP
**DT**	1	-	-	-	-	-
**Imag**	-.204**	1	-	-	-	-
**Perse**	.001	.311**	1	-	-	-
**Insight**	-.086	.393**	.501**	1	-	-
**Explo**	-.152*	.430**	.294**	.279**	1	-
**Total CP**	-.155*	.802**	.732**	.723**	.615**	1

**p<0.01;

*p<0.*05* (two-tailed test).

**Table 6:** Correlations between dialectical thinking and different dimensions of creative personality: UMO group (*N* = 203).

	DT	Imag	Perse	Insight	Explo	Total CP
**DT**	1	-	-	-	-	-
**Imag**	.067	1	-	-	-	-
**Perse**	-.012	.287**	1	-	-	-
**Insight**	-.016	.528**	.427**	1	-	-
**Explo**	.046	.654**	.355**	.424**	1	-
**Total CP**	.032	.839**	.681**	.752**	.777**	1

**p<0.01;

*p<0.*05* (two-tailed test).

According to Table 3, significant positive correlations among the four dimensions and total score of creative personality were found. Regarding the relationship between dialectical thinking and creative personality, significant but weak negative correlations were found among dialectical thinking and imagination, insight, and the total score of creative personality, no other significant correlation was found. These results suggested that participants scoring higher on dialectical thinking would tend to score lower on imaginative and insightful personalities.

Similar to the results of Table 3, in all three participant groups, stable positive correlations were found within the four dimensions of creative personality (see Tables 4, 5, and 6). Though these positive relationships indicate a good construct validity of the creative personality scale, the relationship between dialectical thinking and creative personality was rather complicated across the different groups. Tables 4 and 5 showed somehow different pattern for the relationship between dialectical thinking and creative personality in the UMA and AG groups. The familiar part of these two relationship is only exist in the relationship between dialectical thinking and imagination and the total score of creative personality, which both show a significant but weak negative correlation. That means the higher the level of dialectical thinking, the lower the score of imagination and the total score of creative personality. Furthermore, except these relationships, all of other correlations are different between the two groups. In the UMA group, dialectical thinking also has significant negative but weak correlation with perseverance and insight. Nevertheless table 5 did not show the same correlations, and dialectical thinking only has a significant negative but weak correlation with exploration. By contrast, the results in Table 6 showed that compared with the art students or the adults working in arts-related professions, undergraduates from other disciplines showed more dialectical thinking (see Table 1), but no significant correlation was found between their dialectical thinking and creative personality.

Based on these correlation results, to further excavate the association between dialectical thinking and creative personality, a regression analysis was conducted with the total creative personality score and the scores of its four dimensions as the dependent variables. Due to the significant difference in the dialectical thinking scores among the three groups (see Tables 1 and 2), Tables 7, 8, and 9 individually present the results of the regression analysis for each of the three sample groups.

**Table 7:** Results of the regression analysis with creative personality as a dependent variable: UMA group (*N* = 84).

Dependent Variable	*R*	*R* ^*2*^	*F*	*β*
**Imagination**	.273	.075	6.603*	-.273*
**Perseverance**	.282	.080	7.097**	-.282**
**Insight**	.323	.104	9.564**	-.323**
**Exploration**	.151	.023	1.915	-.151
**Total CP**	.374	.140	13.295**	-.374**

Note. All decimals are rounded up to three digits. *β* is the standardized regression coefficient (the same definition is used in subsequent tables)

**p<0.01;

*p<0.*05* (two-tailed test).

**Table 8:** Results of the regression analysis with creative personality as a dependent variable: AG group (*N* = 202).

Dependent Variable	*R*	*R* ^*2*^	*F*	*β*
**Imagination**	.204	.041	8.643**	-.204**
**Perseverance**	.001	.000	.000	-.001
**Insight**	.086	.007	1.495	-.086
**Exploration**	.152	.023	4.731*	-.152*
**Total CP**	.155	.024	4.910*	-.155*

**p<0.01;

*p<0.*05* (two-tailed test).

**Table 9:** Results of the regression analysis with creative personality as a dependent variable: UMO group (*N* = 203).

Dependent Variable	*R*	*R* ^*2*^	*F*	*β*
**Imagination**	.067	.005	.909	.067
**Perseverance**	.012	.000	.030	-.012
**Insight**	.016	.000	.052	-.016
**Exploration**	.046	.002	.431	.046
**Total CP**	.032	.001	.207	.032

**p<0.01;

*p<0.*05* (two-tailed test).

As shown in Table 7, in the UMA group, dialectical thinking accounted for different degrees of variance in the measure of creative personality, except in the dimension of exploration. Dialectical thinking accounted for 7.5% of the variance in the measure of imagination, 8% in the measure of perseverance, 10.4% in the measure of insight, and 14% in the measure of the total score of creative personality.

As shown in Table 8, in the AG group, the explanatory power of dialectical thinking decreased. Dialectical thinking only significantly accounted for the variance of imagination (4.1%, *β* = -0.204, *p*<0.01), the variance of exploration (2.3%, *β* = -0.152, *p*<0.05), and the variance of the total score of creative personality (2.4%, *β* = -0.155, *p*<0.05). All of the *R* ^*2*^ were lower than 5%. These results indicated that, in this group, dialectical thinking was less related with creative personality than in the UMA group.

For the UMO group (Table 9), no significant standardized coefficient of *β* was found. These results indicated that their dialectical thinking had no relationship with creative personality.

## Discussion

In this study, a questionnaire survey method was used to investigate the relationship between dialectical thinking, a typical representation of East Asian culture, and creative personality. The consideration of this relationship is not a new study focus or research trend. In the past several decades, Dean Simonton has devoted himself to the investigation of eminent creators in human history [26] [28] [57] [58]. All of his research findings have suggested that the social environment (including cultural conditions) can affect the development of creativity. Nakamura and Csikszentmihalyi proposed a model of cultural evolution concerning creativity [59]. They do not regard creativity as a quality or a product of exclusively intrapsychic processes. Instead, they view it as the transformation of a cultural system—the incorporation of novelty into the culture (see [30], [59], [60], [61]). As an exemplar of collectivism in East Asian culture, the Chinese typically show a dialectical and holistic thinking style. Although their thinking style basically differentiates them from people living in Western cultures, there is no lack of highly creative persons in either culture. Therefore, this phenomenon raises several questions: What is the relationship between East Asian culture and creativity? Why is Chinese culture, which is different in many aspects from Western culture, also conducive to creativity? Does dialectical thinking have a special influence on creativity or creative personality? To answer these questions, this study aimed to explore the relationship between dialectical thinking style and creativity, especially the creative personality of Chinese people. In other words, in Chinese culture, we aimed to learn whether people with a creative personality would simultaneously show a dialectical thinking style. Two questionnaires, one that surveyed creative personality and another that assessed dialectical thinking style, were administered in our study, drawing interesting and valuable results.

### The relationship between creative personality and dialectical thinking

According to Tables 1 and 2, no significant difference was found between undergraduates majoring in art and adults working in an arts-related field in terms of their creative personality. However, the third group of participants, the undergraduates from other disciplines, had the lowest scores on creative personality by a significant margin. All of our participants are Chinese, so why did they show obvious discrepancies in creative personality? The main reason may come from the domains in which they study and work. Art and design as specialties share many characteristics, such as divergent thinking [62] [63] and rule breaking [13]. However, the differences between these two areas and other inartistic disciplines are easy to distinguish. Therefore, the distinction between these groups can be understood. In general, this finding is in line with previous studies, which have indicated that the relationship between creativity and dialectical thinking is culture-, task-, and process-specific [42]. In terms of the influencing factors of creative personality, given that the participants were all Chinese, this result may indicates that besides the culture influence, participants were also affected by their work or specialty additionally. Moreover, this finding is consistent with Hass’s conclusions that laypersons’ implicit theory of creativity is domain-specific [64], although in Hass’s study, the creative traits were rated by others.

Another important result that we have drawn from the analysis of the descriptive statistics is the differences in dialectical thinking. Our results showed that, contrary to the results for creative personality, the highest dialectical thinking score was found in the UMO group, followed by the AG group, and the UMA group had the lowest dialectical thinking score. As we noted above, creative personality is affected by their major or specialty, and so is dialectical thinking. According to Peng and Nisbett [38], dialectical thinking seeks to retain basic elements of opposing perspectives by seeking a “middle way”. College students majoring in art and adults work in arts-related professions usually need to retain existing or old characteristics or expressions [65]. However, the outcomes of their art or design work must be refreshed, renewed, and reinterpreted [65] [66], which we consider to be a more distinct and “noncontradiction” way of finding solutions. Based on this understanding, college students majoring in other disciplines will have relatively more practice in dialectical thinking. Thus, they received higher scores on dialectical thinking than the other two groups. Nonetheless, the difference between the other two groups may have more to do with participants’ cultural background. In other words, thinking style may not *only* rely on the impact of domain. Regarding the influence of culture on dialectical thinking, though the Chinese culture fosters a holistic and dialectical thinking style, it is not the same as it is in the West. Western dialectical thinking often reflects a sophisticated and advanced cognitive style that attempts to achieve syntheses over contradiction [67] [68], while the East Asian type of dialectical thinking is less troubled by contradiction and accepts the coexistence of opposites, even the unity of thesis and antithesis [38]. According to this view, dialectical thinking is culture-specific rather than culture-free. On the other hand, from a developmental perspective, this culture-specific trait also helps to explain the significant difference in dialectical thinking between the UMA and AG groups. Obviously, with an increase in age and experience, Chinese people will more frequently confront and experience Chinese traditional philosophy and wisdom in their daily lives, and these ubiquitous phenomena will further foster and stabilize their naive dialectical thinking style. As more mature dialectical thinkers, adults working in arts-related fields will show more agreement with dialectical thinking than undergraduates who major in a similar specialty. These understandings are also helpful for the explanation of why the UMA group had the lowest score on dialectical thinking of the three groups. Combining the group differences of creative personality and dialectical thinking, Hypothesis 1 of this study was confirmed. The two main variables in our study can be differentiated according to their major and job.

The third important finding was the significant, but meantime partial and weak negative relationship between creative personality and dialectical thinking in the UMA and AG groups. Meanwhile, no significant relationship between these two variables was found in the UMO group. First, this result contradicts Arlin and Levitt’s finding, which indicated a positive relationship between dialectical thinking and problem finding [45]. However, the differences in these two studies are easy to understand. One difference is that the concepts and instruments of dialectical thinking used in these two studies are heterogeneous. Arlin and Levitt’s study used the concept of traditional Western dialectical thinking, which is a representation of formal logic and formal reasoning. The key feature of this thinking style is integration [38] [45]. In Eastern culture, dialectical thinking is also called “naive dialecticism”. It has three key principles, which were noted above, and these principles are distinct from their counterparts in Western culture [38]. The other difference is the measure of creativity. Arlin and Levitt’s study collected data from participants’ cognitive problem finding results [45], whereas in this study, we measured the creative personality of the respondents. Based on these two reasons, the differences of the results are expectable. Second, our findings are in line with Paletz and Peng’s study, which found that naive dialectical thinking and creativity in a problem finding task were negatively related [38]. In their study, the relationship was significant only for Caucasians and not for Asians/Asian-Americans. In other words, that study confirmed the negative relationship in Western culture, and the current study confirmed this relationship in an East Asian culture. Third, in another cross-cultural study, as noted above, Yi et al. compared the performances of artistic creativity of German and Chinese college students. The results revealed that the four groups of students examined (German students of Caucasian descent, German students of Asian descent, Chinese students studying abroad, and Chinese students studying in China) differed in their artistic creativity. German students showed more artistic creativity than their Chinese counterparts [37]. Therefore, the current study provides more evidence for the role played by a specific culture in terms of the relationship between thinking style and creative personality. Moreover, our findings are consistent with the realities of everyday life in China. As a matter of fact, the majority of Chinese people are used to the naive dialectical thinking style, and relatively little creative style is shown in their thinking and behavioral processes, especially because they emphasize their interpersonal relationships, the collectives to which they belong, and the feelings of others. To summarize, a person who is characterized by a more dialectical thinking style will simultaneously show less characteristics of imagination and insight personalities. Essentially, Hypothesis 2 of our study was partially confirmed.

### Why did less-dialectical thinking style negatively relate with creative personality?

In this study, college students majoring in art and adults working in arts-related fields received relatively lower scores on dialectical thinking than undergraduates majoring in other disciplines, which indicated they think in a less-dialectical way. Based on the results of this study and the discussion above, an important theoretical question was raised: Why does a less-dialectical thinking style negatively related with creative personality? Three different aspects provide an answer.

The first is the aspect of cultural differences. Triandis indicated that some cultures, such as those of Western Europe and North America, rated highly on individualism [33]. In these cultures, the self is defined as independent and autonomous from collectives. Personal goals are given priority over the goals of collectives. Individualists give more weight to attitudes than to norms as determinants of social behavior [69–71], and personal creativity is highly appreciated. Unlike these cultures, cultures that highlight collectivism, for example, East Asian culture, define the self as an aspect of a collective (e.g., a family or tribe). In these cultures, personal goals are subordinated to the goals of their collective; norms, duties, responsibilities, and obligations regulate most social behavior, and taking into account the needs of others in the regulation of social behavior is widely practiced. People in these cultures appreciate norms, obedience, cooperation, and compliance with authority. From these analyses of cultural differences, one may figure out that Western culture encourages creativity more than East Asian culture does.

Second, with respect to the nature of dialectical thinking, it is not only an alternative social cognitive and thinking style but also a fundamental component of Chinese holistic thinking and the zhong-yong thinking style. In recent years, a research trend on the concept of zhong-yong thinking and its role in interpersonal and intrapersonal psychological processes has emerged in social and cultural psychological research. Mainly using Chinese subjects, several studies have agreed that the nature of zhong-yong thinking was an effective strategy for keeping interpersonal concordance; it improved interpersonal cooperation and made people adopt more cooperative behaviors in conflict situations [72] [73]. Zhong-yong thinking also played a positive role in the regulation of personal emotions [74] [75]. Wu [76] and Liu [77] also found that zhong-yong thinking was positively related with several positive emotional and mental factors, such as life satisfaction, subjective well-being, and trust, but had a negative relationship with depression, anxiety, and other negative emotions. In summary, zhong-yong thinking resembles dialectical thinking in that both entail maintaining balance or finding a “middle way” between different solutions. This approach is not consistent with the traits of creativity, which involve breaking rules [13], finding novel solutions [1], and so on. Thus, according to the previous studies about zhong-yong thinking style [72–77] and our research findings, both zhong-yong thinking and dialectical thinking have partial and weak negative relationships with creative personality.

### What constitute creativity in East Asian cultures?

One may be confused, based on the “naive dialecticism” thinking style rooted in East Asian cultures, and also based on the weak and negative correlations between dialectical thinking and some traits of creative personalities, why still Chinese people performed a marked level of creativity in their splendid history? Whether Chinese are creative enough to handle the important innovative problems in the field of art or science today? And the most important and interesting, different from western culture, what constitute creativity in China and East Asian cultures? We think there are two main explanations. First, Chinese people’s creativity may embodied in different aspects of creative ability from western cultures. About the category of creativity, some researchers [78] proposed a four C model. This model views creativity in four categories: Big-C (eminent, genius-level), Pro-c (professional-level), little-c (everyday creativity), and mini-c (personal, expressive, fluid, and developmental aspects of creativity) [79] [80]. Like we mentioned before, the Big-C has been measured historiometrically [81]. This is also suitable for the measure of Chinese creativity, especially in their brilliant history. Nevertheless, today we define creativity as an ability to generate novel ideas [3]. We always test it through a cognitive approach [11], and many kind of divergent thinking tests and creative personality scales have been developed. Obviously, these measurements are different from the historiometrical way of measurement for Chinese creativity. So we cannot compare the evidences directly which were found through different methods.

Second, Chinese may show more creative traits in a cognitive way than in the personality facet. As we mentioned above, in Chinese people’s everyday life, they care more about the collectives to which they belong, and the feelings of others than themselves. But creative ideas are often deviant [82], expressing this kind of thought are at-risk of receiving negative evaluations from other group members [83]. It is also harmful for the harmony with others and the collectives. So little creative personalities or behaviors are showed when one stay in the collectivism groups. Even so, Chinese are still competitive when their ability of creative thinking was compared with western counterparts. Previous study found that, compared with adolescents in England, Chinese adolescents show superior creative problem solving ability to that of English adolescents [35]. In other words, in Chinese and East Asian cultures, creativity must be approached as a dichotomy. Then a conclusion can be drawn: people live in East Asian cultures show little creative personalities, but it may accompany with a relatively higher level of ability on creative cognitive processing. In the present study, we only collected data about creative personality. Future research may extend the scope of creativity from personality to cognitive factors.

### Limitations and Suggestions for Future Research

Due to the growing body of dialectical thinking research and the complexity of creativity, this study had a few weaknesses, and these weaknesses could be overcome by additional research. First, though this study sampled subjects from different cities and different disciplines and professions, the domain they were engaged in was nonetheless limited to artistic creativity. Therefore, the sampling process caused a problem of external validity. People who are engaged in and have opportunities to show their scientific creativity should be sampled in future research. Through this approach, researchers could broaden the diversity and representativeness of the subjects so that the research data have better generalizability.

Second, the measurement of dialectical thinking in this study consisted only in the use of a questionnaire. Due to the universality and popularity of the dialectical thinking style in Chinese culture, the respondents could be affected by social desirability during the responding process. Therefore, future research should adopt a diverse research paradigm such as a laboratory experiment design to collect data on dialectical thinking. Using a different data collection approach, for example, priming, and the social desirability effect may be effectively reduced.

Third, the results of this study were often weak. This may be due to a lack of counterpart participants. The lack lies in two aspects. First, we sampled undergraduates majoring in art and in non-art disciplines, but we only recruited one group of adults who work in art-related field. There is no paralleled adult group to the undergraduates majoring in other disciplines. Second, we tested Chinese people’s dialectical thinking style, but what about people living in Western countries and those familiar with Western culture? To what extent will they think in a dialectical way? Answering these questions would be useful to better understand the relationship between dialectical thinking and creative personality in a developmental way, and in both Western and East Asian cultures. Researchers in future study should be more deliberate before they recruit participants, and carefully compare their data to provide more powerful evidences.

## Conclusion

Starting with the definition of dialectical thinking, we have investigated the differences in dialectical thinking style among different groups of Chinese people. The results indicated that people engaged in different fields showed different levels of dialectical thinking. We have also explored the relationship between their creative personality and dialectical thinking. Interestingly, we have found that, in Chinese culture, people, especially the undergraduates majoring in art, who showed a dialectical thinking style, may have low levels of personality traits on imagination and insight.

These findings can improve our understanding of creative personality and the influence that thinking style, work and specialty may have on it. In other words, these factors make additional contributions besides culture. Moreover, we knew that dialectical thinking style is incompatible with creative personality to some degree in East Asian cultures. To cultivate creative personality in Chinese college students and adults, a less-dialectical thinking style should be encouraged and taught to a certain extent. Especially in those disciplines and specialties which need more creative personality and thinking style than others.

## Supporting Information

S1 Dataset (SAV)
